# A single phenylalanine residue in β-arrestin2 critically regulates its binding to G protein–coupled receptors

**DOI:** 10.1016/j.jbc.2022.101837

**Published:** 2022-03-17

**Authors:** Pierre-Yves Jean-Charles, Vishwaesh Rajiv, Subhodeep Sarker, Sangoh Han, Yushi Bai, Ali Masoudi, Sudha K. Shenoy

**Affiliations:** 1 Division of Cardiology, Department of Medicine, Duke University Medical Center, Durham, North Carolina, USA; 2 Department of Cell Biology, Duke University Medical Center, Durham, North Carolina, USA

**Keywords:** arrestin-domain containg proteins, trafficking, ubiquitination, K63 chain, arrestin N-domain, AngII, angiotensin II, β-arr2, β-arrestin2, β_2_AR, β_2_-adrenergic receptor, ART, arrestin-related trafficking adaptor, Art1, arrestin–related trafficking protein 1, AT_1a_R, angiotensin II type 1a receptor, CHX, cycloheximide, co-IP, coimmunoprecipitation, DPBS, Dulbecco's PBS, DSP, dithiobis(succinimidyl propionate), ERK, extracellular signal–regulated kinase, GPCR, G protein–coupled receptor, HA, hemagglutinin, HEPES, 4-(2-hydroxyethyl)-1-piperazineethanesulfonic acid, HBSS, Hanks' balanced salt solution, HEK-293, human embryonic kidney 293 cell line, HRP, horseradish peroxidase, IBMX, 3-isobutyl-1-methylxanthine, IgG, immunoglobulin, K48, lysine 48, K63, lysine 63, MAPK, mitogen-activated protein kinase, MDM2, mouse double minute 2, NP-40, Nonidet P-40, PDB, Protein Data Bank, PDE, phosphodiesterase, TRAF6, tumor necrosis factor receptor–associated factor 6, Ub, ubiquitin, USP20, ubiquitin-specific peptidase 20, Vps26, vacuolar protein sorting–associated protein 26, V2R, vasopressin V2 receptor

## Abstract

Arrestins and their yeast homologs, arrestin-related trafficking adaptors (ARTs), share a stretch of 29 amino acids called the ART motif. However, the functionality of that motif is unknown. We now report that deleting this motif prevents agonist-induced ubiquitination of β-arrestin2 (β-arr2) and blocks its association with activated G protein–coupled receptors (GPCRs). Within the ART motif, we have identified a conserved phenylalanine residue, Phe116, that is critical for the formation of β-arr2–GPCR complexes. β-arr2 Phe116Ala mutant has negligible effect on blunting β_2_-adrenergic receptor–induced cAMP generation unlike β-arr2, which promotes rapid desensitization. Furthermore, available structures for inactive and inositol hexakisphosphate 6–activated forms of bovine β-arr2 revealed that Phe116 is ensconced in a hydrophobic pocket, whereas the adjacent Phe117 and Phe118 residues are not. Mutagenesis of Phe117 and Phe118, but not Phe116, preserves GPCR interaction of β-arr2. Surprisingly, Phe116 is dispensable for the association of β-arr2 with its non-GPCR partners. β-arr2 Phe116Ala mutant presents a significantly reduced protein half-life compared with β-arr2 and undergoes constitutive Lys-48-linked polyubiquitination, which tags proteins for proteasomal degradation. We also found that Phe116 is critical for agonist-dependent β-arr2 ubiquitination with Lys-63-polyubiquitin linkages that are known mediators of protein scaffolding and signal transduction. Finally, we have shown that β-arr2 Phe116Ala interaction with activated β_2_-adrenergic receptor can be rescued with an in-frame fusion of ubiquitin. Taken together, we conclude that Phe116 preserves structural stability of β-arr2, regulates the formation of β-arr2–GPCR complexes that inhibit G protein signaling, and promotes subsequent ubiquitin-dependent β-arr2 localization and trafficking.

Arrestins, which include visual arrestins (arrestin 1 and arrestin 4 in rods and cones, respectively) and ubiquitously expressed nonvisual arrestins (arrestin 2 and arrestin 3, also termed β-arrestin1 or β-arr1 and β-arr2, correspondingly) are polyfunctional cytosolic adaptor proteins, typically known for their ability to inhibit cellular increase in second messengers resulting from the agonist stimulation of G protein–coupled receptors (GPCRs) (1, 2). GPCRs, also called seven transmembrane receptors, are a diverse group of heptahelical membrane proteins that couple to heterotrimeric guanine nucleotide–binding proteins (G proteins) to initiate cellular response to ligand stimulation (3, 4). Arrestin inhibition of GPCR signaling requires two mechanisms: (1) phosphorylation of the C-terminal tail of the stimulated receptors by GPCR kinases and (2) recruitment and binding of arrestin to the phosphorylated tail and transmembrane core of the receptor for steric hindrance of G protein coupling and activation (1, 5, 6). In addition to inhibiting G protein coupling (a process termed receptor desensitization), β-arrs regulate other aspects of the fate and activity of GPCRs such as (1) receptor internalization by recruiting components of the endocytic machinery, clathrin, and β-adaptin subunits, (2) receptor ubiquitination by serving as adaptors for ubiquitin (Ub) ligases and/or deubiquitinases, and (3) signal transduction by scaffolding various kinases and nonkinase proteins into signaling modules (1, 7, 8, 9, 10, 11, 12, 13). The multifaceted roles of β-arrs in regulating GPCR functions are directed by their ability to embrace various conformations, which are dictated by the phosphorylation profile of the activated GPCR tail and/or by agonist-induced post-translational modifications of β-arrs, which altogether contribute to the kinetics and stability of receptor–β-arr interaction (13, 14, 15, 16).

β-arr interaction with activated GPCRs involves its binding to the phosphorylated tail of the receptor and may not involve full integration of β-arr protein domains into the transmembrane core of the receptor, resulting in the formation of distinct GPCR–β-arr complexes: fully engaged β-arr (core conformation) or partially engaged β-arr (tail conformation) with β-arr hanging on the receptor's tail (17, 18). While the core conformation is essential for steric hindrance of G protein coupling, the tail conformation is sufficient for β-arr-mediated receptor endocytosis, β-arr scaffolding of various components of mitogen-activated protein kinase (MAPK) cascades such as extracellular signal–regulated kinase (ERK) 1 and 2, as well as enabling GPCR, G protein, and β-arr supercomplex formation for sustained signaling in endosomes (17, 19, 20, 21).

Structurally, arrestins consist of two modules of antiparallel β-sheets: an N-terminal and a C-terminal domain that are connected by a flexible linker region (22, 23, 24, 25). This structure known as the “arrestin fold” is conserved between arrestin isoforms and between species. The arrestin fold is also present in other cytosolic proteins that share functional kinship with arrestins, notably in regulating trafficking processes and/or in mediating receptor ubiquitination (26). The arrestin fold–containing proteins found in humans include arrestin domain–containing proteins 1 to 5 and thioredoxin-interacting protein, which are collectively known as α-arrestins. Additional proteins distantly related to arrestins that possess the arrestin fold include vacuolar protein sorting–associated protein 26 (Vps26), Vps26-related protein Down syndrome critical region gene 3, and retrograde Golgi transport protein 1 (27). In *Saccharomyces cerevisiae*, the arrestin fold is present in a family of proteins termed arrestin-related trafficking adaptors (ARTs). Yeast arrestin-like proteins include Art 1 to 10 as well as the fungus-specific Art variants, Bul 1 to 3 (27, 28, 29, 30).

Despite presenting structural and some functional similarities with β-arrs, arrestin fold–containing proteins share minimal sequence homology with arrestins and lack several characteristic features of arrestins. For example, Vps26 lacks GPCR, clathrin, and phosphatidylinositol phospholipid-binding sites that are typically present in the visual arrestins and β-arrs (27). In addition, ART proteins and α-arrestins lack the N domain helix that is found in arrestins, whereas their C-tails, which do not contain clathrin-binding site, present PPxY motifs (except for arrestin domain–containing protein 5), which allow binding to WW domain–containing proteins, such as E3 Ub ligases of the Rsp5p/Nedd4 family (27, 28, 31, 32). Nevertheless, β-arrs and their yeast homologs do share a short stretch of 29 amino acid sequence called arrestin/ART motif (28). The ART motif is highly conserved, which suggests that it plays an important role in the function of these proteins. However, the role of the ART motif in β-arrs has not been investigated yet. Using site-directed mutagenesis and cell-based assays, we have determined the significance and functional contribution of the ART motif in β-arr2.

## Results

### ART motif in β-arr2 is required for associating with activated GPCRs

**Figure 1:**
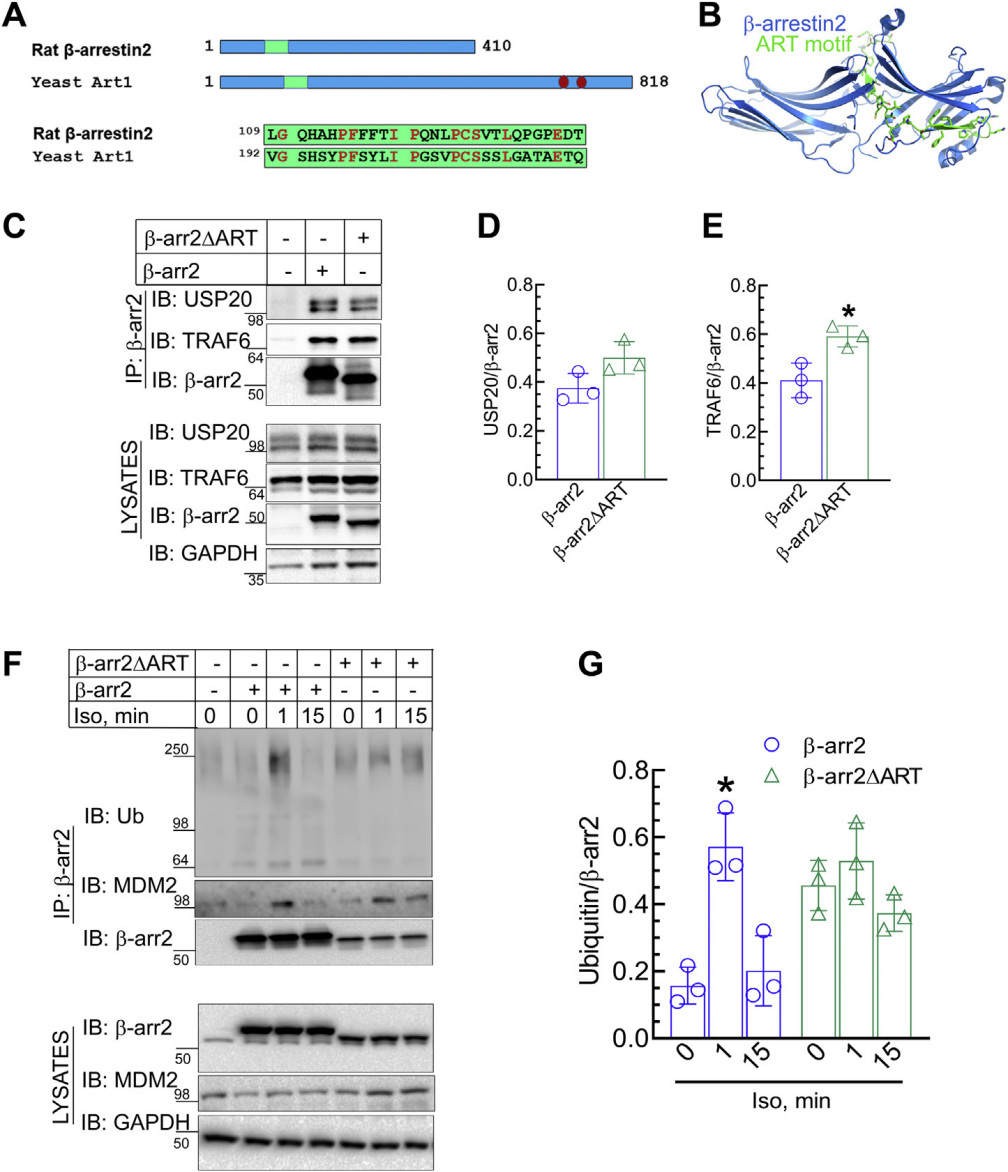
**ART motif in β-arrestin2 (β-arr2) is dispensable for partner protein interactions but required for agonist-induced ubiquitination.***A*, schematic comparing the location and sequence of ART motif in β-arr2 and yeast arrestin-related trafficking protein 1 (Art1). *B*, location of the ART motif shown in *green* traversing across β-arr2 molecule. Structure coordinates from 3P2D were used to generate molecular figure using PyMOL (DeLano, W. L. (2012) *The PyMOL Molecular Graphics System v. 1.3r1*, Schrödinger, LLC, New York, NY). *C*, HA-tagged β-arr2 or β-arr2ΔART expressed in HEK-293 cells was immunoprecipitated, and the associations of endogenous USP20 and TRAF6 were determined by immunoblotting. Lysate blot panels show the expression of USP20, TRAF6, and β-arrs in each sample. Blots presented are from one of three independent experiments. Band signals of USP20 normalized to β-arr2 and of TRAF6 similarly normalized from three independent experiments are summarized as means ± SD in the scatter plots with bars in (*D*) and (*E*), respectively. ∗*p* < 0.05, unpaired *t* test. *F*, HA-tagged β-arr2 or β-arr2ΔART was isolated by HA-affinity pull down and immunoblotted for ubiquitin (FK2), MDM2, and β-arr2. Lysates were serially probed for HA, MDM2, and GAPDH as loading control. *G*, ubiquitin signals in (*F*) were quantitated and normalized to cognate β-arr2 or β-arr2ΔART and summarized as scatter plots with bars from three independent experiments. ∗*p*< 0.05 *versus* 0′ and 15′ samples; two-way ANOVA, Holm–Sidak's test. ART, arrestin-related trafficking adaptor; HA, hemagglutinin; HEK-293, human embryonic kidney 293 cell line; MDM2, mouse double minute 2; TRAF6, tumor necrosis factor receptor–associated factor 6; USP20, ubiquitin-specific peptidase 20.

To define the characteristics of the ART motif, we examined conservation of amino acid residues in that region by performing sequence alignments of β-arr2 and yeast arrestin–related trafficking protein 1 (Art1) (Fig. 1*A*). While the ART motif in rat β-arr2 was not identical with that of yeast Art1, a substantial number of conserved residues were identified in rat, bovine, human, and mouse β-arr2 and include Gly110, His112, Pro115, Phe116, Ile120, Pro121, Pro125, Cys126, Ser127, Leu130, and Glu135 (Figs. 1*A* and 3*A*). The ART motif (*green*, Fig. 1, *A* and *B*) is located in the N domain of β-arr2, traversing across the β-arr2 molecule and linking both N and C domains of β-arr2 (Fig. 1*B*).

We next determined if the ART motif affects the interaction of β-arr2 with its binding partners. β-arr2 is a multifunctional adaptor protein that not only regulates GPCR signaling but also associates with other types of membrane receptors, as well as nuclear receptors, ions channels, and various cytosolic signaling mediators to regulate a variety of cellular activities (1, 2, 12, 33). Accordingly, the binding partners of β-arrs are diverse and, besides GPCRs, include kinases and Ub pathway enzymes such as the E3 Ub ligase tumor necrosis factor receptor–associated factor 6 (TRAF6) and the deubiquitinating enzyme ubiquitin-specific peptidase 20 (USP20), which form a tripartite complex with β-arr2 (34). The interaction of β-arr2 with USP20 and TRAF6 prevents autoubiquitination of TRAF6, inhibiting NF-κB activation and inflammatory signaling downstream the Toll-like receptor 4 (34). Deletion of the entire ART motif did not prevent expression of exogenous β-arr2 in human embryonic kidney 293 (HEK-293) cells. Nonetheless equivalent DNA transfection in our cell-based assays yielded lesser expression of the ART motif–deleted mutant protein β-arr2ΔART compared with overexpressed WT β-arr2. In our pull-down assays, we observed that as for the WT β-arr2, both TRAF6 and USP20 coimmunoprecipitated with the β-arr2ΔART mutant (Fig. 1, *C*–*E*). Moreover, while USP20 showed an increasing trend of protein association with β-arr2ΔART, TRAF6 binding to β-arr2ΔART was significantly higher when compared with WT β-arr2 (Fig. 1, *D* and *E*). Accordingly, the ART motif might be dispensable for the protein interactions of β-arr2, and the scaffolding properties of β-arr2 may be negatively regulated by the ART motif.

Next, we determined the effect of the ART motif on β-arr2 ubiquitination. Ubiquitination is a reversible post-translational modification in which the 76-amino acid protein Ub is appended to substrate proteins, tagging them for various cellular activities, such as degradation, vesicular trafficking, protein scaffolding, and cell signaling (16, 35, 36, 37, 38). Ubiquitination of β-arr2 was first identified for the β_2_-adrenergic receptor (β_2_AR) pathway and is mediated by mouse double minute 2 (MDM2), an oncogenic E3 Ub ligase primarily known for inhibiting the proapoptotic/tumor-suppressor protein p53 (14, 39). β-arr2 ubiquitination by MDM2 occurs within minutes of the recruitment of β-arr2 to the activated β_2_AR and is necessary for subsequent activity of β-arr2 such as stabilization of the receptor interaction with β-arr, greater binding affinity with clathrin subunits to engage receptor endocytosis, and tighter association of β-arr2 with components of MAPKs such as c-RAF and ERK for endosomal signaling (12, 40). Using HEK-293 cells stably expressing either β-arr2 WT or β-arr2ΔART mutant, we stimulated β_2_AR with the agonist isoproterenol and monitored β-arr2 ubiquitination using an antibody that recognizes total polyubiquitin chains. In cells expressing exogenous WT β-arr2, 1 min stimulation with isoproterenol induced a significant increase in ubiquitination of β-arr2 than at the basal state (Fig. 1, *F* and *G*, Ub:β-arr2, 0.15 ± 0.03 at basal *versus* 0.57 ± 0.05 at 1′ iso treatment). On the other hand, the β-arr2ΔART mutant was constitutively ubiquitinated at a higher level than WT β-arr2, and agonist stimulation of the β_2_AR failed to increase the ubiquitination status of β-arr2ΔART mutant (Fig. 1, *F* and *G*). Notably, the association of MDM2 and β-arr2 was mostly unaffected by the ART motif deletion (Fig. 1*F*). Our results reveal that the ART motif is not required for protein interaction with MDM2 but essential for agonist-dependent ubiquitination of β-arr2 upon β_2_AR activation.

**Figure 2:**
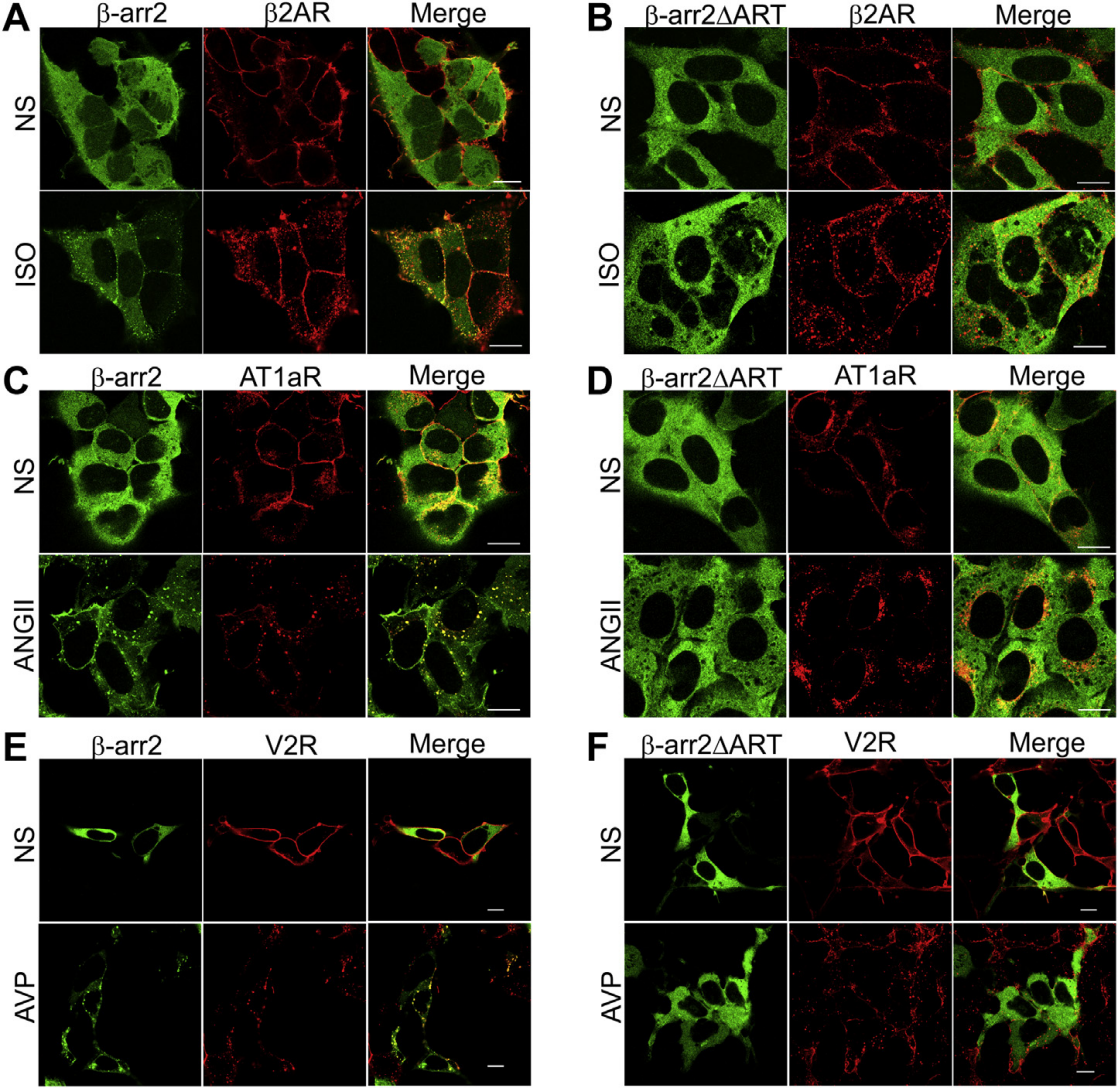
**ART motif in β-arrestin2 (β-arr2) is required forinteractingwith activated G protein–coupled receptors**. HEK-293 cells stably expressing β_2_AR were transfected with YFP-βarr2 (*A*) or YFP-β-arr2ΔART (*B*) and stimulated ±1 μM isoproterenol (Iso) for 5 min and processed for confocal imaging (see the Experimental procedures section). Representative images from one of three separate experiments are shown for unstimulated cells (NS) and Iso-stimulated cells for β-arr2 (*green*), β_2_AR (*red*), and merge (*red* and *green channels*). Distribution of YFP-β-arr2 (*C*) and YFP-β-arr2ΔART (*D*) and the angiotensin II type 1a receptor (AT_1a_R, *red*) in NS and angiotensin II (ANG II) stimulated conditions (1 μM, 5 min). Images are from one of three independent experiments. Distribution of YFP-β-arr2 (*E*) and YFP-β-arr2ΔART (*F*) and the vasopressin V2 receptor (V2R, *red*) in NS and arginine–vasopressin (AVP) stimulated conditions (1 μM, 5 min). Images are from one of three independent experiments. The scale bar represents 10 μm. ART, arrestin-related trafficking adaptor; β_2_AR, β_2_-adrenergic receptor; HEK-293, human embryonic kidney 293 cell line.

Since β-arr2 ubiquitination by MDM2 is dependent on the binding of β-arrs to activated β_2_AR, we investigated whether deletion of the ART motif affected the recruitment of β-arr2 to the activated β_2_AR. To assess this, we transfected HEK-293 cells stably expressing FLAG-tagged β_2_AR with either YFP-β-arr2 or YFP-β-arr2ΔART mutant and tested effects of agonist stimulation on β-arr2 translocation. Confocal imaging showed homogenous cytoplasmic distribution of β-arrs (*green*) in quiescent cells, whereas agonist activation of β_2_AR (*red*) provoked translocation of exogenous WT β-arr2 to the cell membrane and colocalization with the stimulated β_2_AR (*yellow*) (Fig. 2*A*). In contrast, exogenous mutant YFP-β-arr2ΔART failed to either translocate to the cell membrane or colocalize with the stimulated β_2_AR and instead remained diffused in the cytoplasm (Fig. 2*B*). Accordingly, the ART motif is essential for the translocation of β-arr2 to the plasma membrane and its recruitment to the stimulated β_2_AR, enabling subsequent activities of β-arr2 when bound to the activated receptor such as its ubiquitination by MDM2.

While the β_2_AR is a class A GPCR that forms a transient complex with β-arr, class B GPCRs promote sustained GPCR/β-arr complex, as well as durable β-arr ubiquitination (41, 42, 43). To discern if the stronger binding provoked by class B receptors might overcome the defect from the deletion of ART motif, we next investigated by confocal imaging (WT and ART deletion mutant) the recruitment of β-arr2 for class B GPCRs, angiotensin II (AngII) type 1a receptor (AT_1a_R) and vasopressin V2 receptor (V2R). As expected, stimulation of the AT_1__a_R by the agonist AngII promoted persistent interaction of exogenous YFP-β-arr2 (*green*) with the AT_1__a_R (*red*), resulting in the internalization of the receptor–β-arr complex and its location in endocytic vesicles, seen as *yellow* puncta in the merged images (Fig. 2*C*). Interestingly, YFP-β-arr2ΔART mutant (*green*) failed to be recruited to the activated AT_1a_R but remained uniformly distributed in the cytosol despite agonist stimulation of the receptor (Fig. 2*D*). As in the case of the AT_1a_R, we detected no colocalization of YFP-β-arr2ΔART with activated V2R, whereas WT β-arr2 cointernalized with the agonist-activated V2R (Fig. 2, *E* and *F*). Our confocal assays further confirm that the deletion of ART motif prevents the translocation of β-arr2 to the plasma membrane and its association with activated GPCRs.

### Molecular and functional analyses of β-arr ART motif

**Figure 3:**
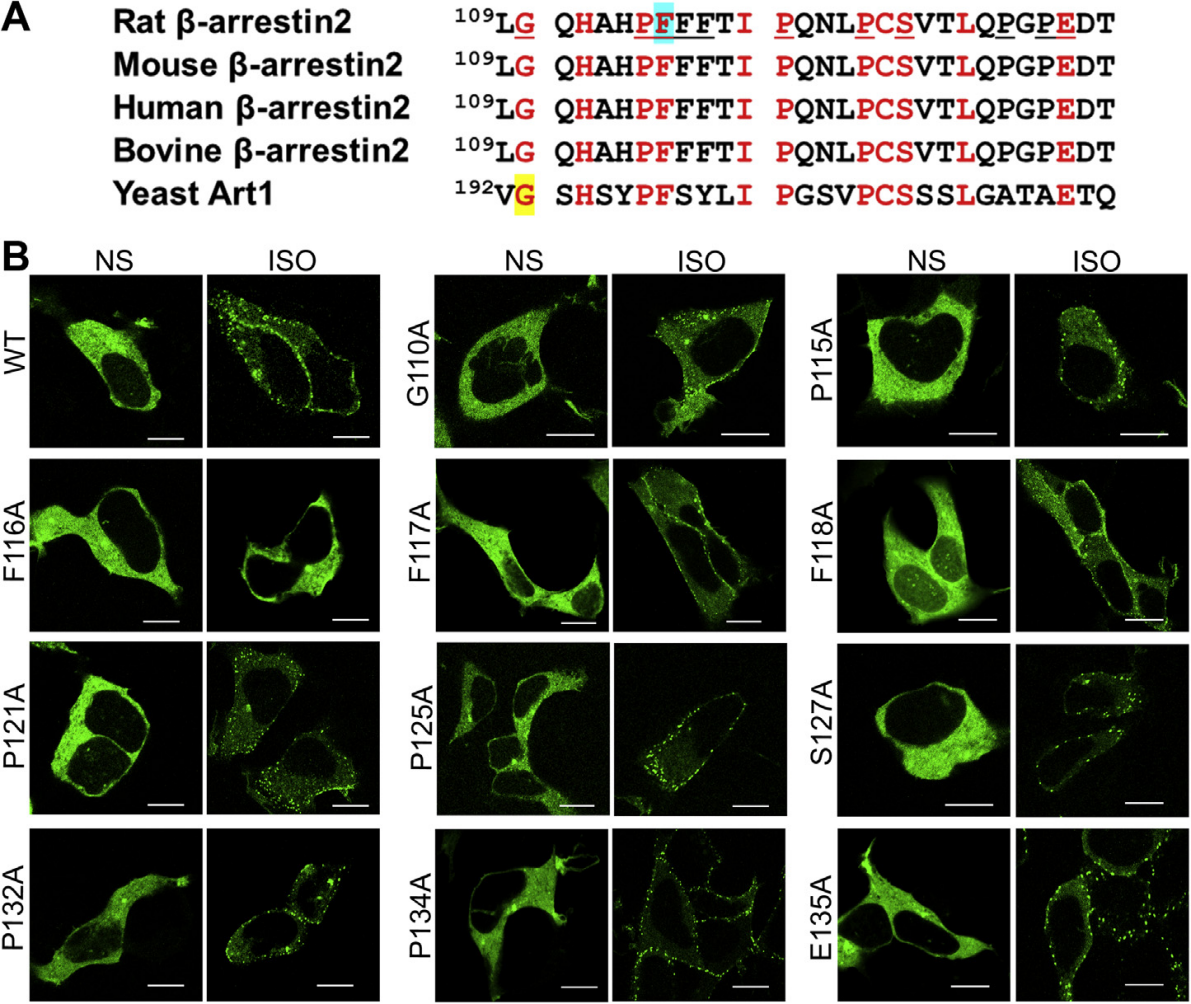
**Assessment of conserved residues oftheARTmotif in β-arrestin2.***A*, amino acid sequence alignment of β-arr2 homologs and yeast Art1 showing conserved residues (*red*) in the ART motif. Underlined residues were tested by mutagenesis in the current study. Critical residues, Gly in yeast Art1 and Phe in rat β-arr2 (this study), are highlighted. *B*, amino acids underlined in rat β-arr2 in *A* were mutated to Ala. YFP-β-arr2 WT and respective mutants were overexpressed in HEK-293 cells stably expressing the β_2_AR. Images of unstimulated and Iso-stimulated cells are shown from one of three independent experiments. The scale bar represents 10 μm. ART, arrestin-related trafficking adaptor; β_2_AR, β_2_-adrenergic receptor; β-arr2, β-arrestin2; HEK-293, human embryonic kidney 293 cell line.

Since the ART motif plays an essential role in the binding of β-arr2 to GPCRs and its subsequent ubiquitination, we looked for the residues in that motif that accounted for its function. Amino acid sequence alignment of β-arr2 homologs and yeast Art1 showed conserved residues in the ART motif such as Gly110, His112, Pro115, Phe116, Ile120, Pro121, Pro125, Cys126, Ser127, Leu130, and Glu135 (Fig. 3*A*). Using site-directed mutagenesis, we replaced each of the aforementioned underlined amino acid residues with alanine. We also generated alanine mutants for Phe117, Phe118, Pro132, and Pro134 that are conserved in arrestins but absent in Art1 (Fig. 3*A*). We next tested the recruitment of these β-arr2 mutants to the stimulated β_2_AR by confocal imaging. Except for the Phe116 residue, mutation of all other conserved amino acid residues preserved β-arr2 (*green*) translocation to the plasma membrane (Fig. 3*B*). In contrast, the β-arr2F116A mutant completely failed to translocate to the plasma membrane after stimulation of the receptor and presented similar homogenous cytoplasmic distribution as in nonstimulated cells, which was the same pattern that we observed for the β-arr2ΔART mutant (Fig. 2).

The two phenylalanine residues adjacent to Phe116, namely, Phe117 and Phe118 (Fig. 3*A*), are conserved in β-arr2 across species, whereas the ART motif of yeast Art1 only has one phenylalanine residue in that corresponding region. To address if the triplet Phe residues played a role in defining the association of β-arr2 with activated GPCRs, we also individually mutated Phe117 and Phe118 to alanine and tested them in GPCR recruitment assays. Interestingly, in contrast to the β-arr2F116A mutant, which completely failed to translocate to the cell membrane, both β-arr2F117A and β-arr2F118A mutations allowed translocation of β-arr2 to the plasma membrane upon β_2_AR activation (Fig. 3*B*). Overall, the effects of the Phe116 residue on β-arr2 recruitment to the activated β_2_AR were distinctive and specific as only Phe116Ala mutation completely obliterated β-arr2 translocation.

**Figure 4:**
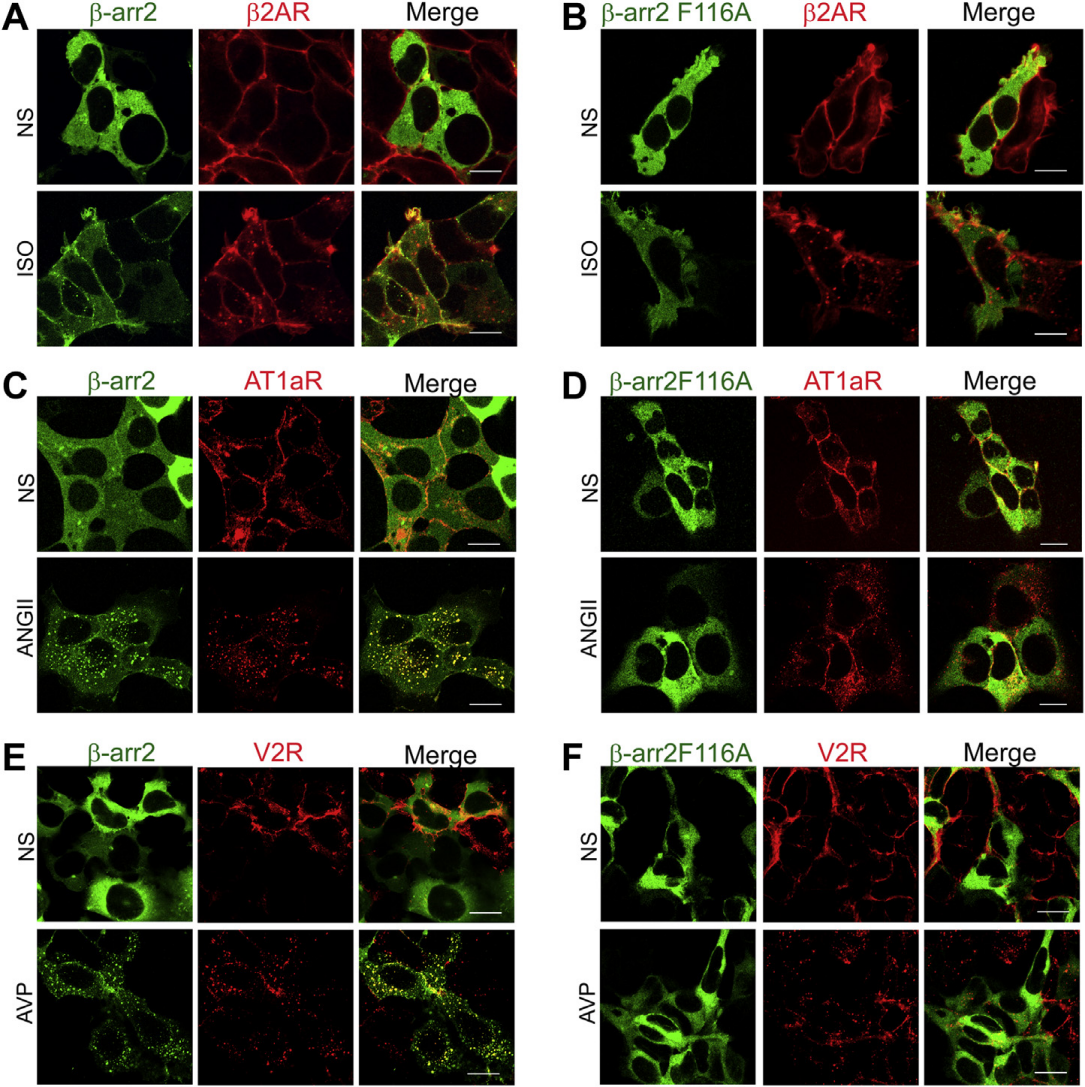
**Phe116 in β-arrestin2 (β-arr2) is required for association with activated G protein–coupled receptors (GPCRs)**. YFP-β-arr2 or YFP-β-arr2F116A was coexpressed with FLAG-β_2_AR (*A* and *B*), HA-AT_1a_R (*C* and *D*), or HA-V2R (*E* and *F*) in HEK-293 cells and stimulated ± respective agonists for 5 min. Representative images from one of three separate experiments are shown for unstimulated cells (NS) and agonist-stimulated cells for β-arr2 (*green*), GPCR (*red*), and merge (*red* and *green channels*). The scale bar represents 10 μm. AT_1a_R, angiotensin II type 1a receptor; β_2_AR, β_2_-adrenergic receptor; HA, hemagglutinin; V2R, vasopressin V2 receptor.

Next, we investigated whether the β-arr2F116A mutant presented similar characteristics as β-arr2ΔART mutant in being recruited to activated β_2_AR, AT_1a_R, and V2R. Unlike exogenous WT β-arr2 (*green*), which was recruited to the activated β_2_AR (*red*), and was colocalized at the plasma membrane with the β_2_AR, visualized as *yellow* puncta at the cell surface (Fig. 4*A*), β-arr2F116A (*green*) failed to be recruited to the stimulated β_2_AR (*red*) but remained diffused in the cytoplasm (Fig. 4*B*). Likewise, WT β-arr2 (*green*) internalized with the activated AT_1a_R (*red*) and colocalized with the receptor in endocytic vesicles (Fig. 4*C*), whereas β-arr2F116A (*green*), similar to the ART motif mutant β-arr2ΔART, did not colocalize with the stimulated AT_1a_R (*red*) in endocytic vesicles, with β-arr2F116A staying uniformly dispersed in the cytosol despite agonist stimulation of the receptor (Fig. 4*D*). β-arr2F116A also failed to associate with agonist-activated V2R unlike WT β-arr2, which cointernalized with the V2R into endocytic vesicles (Fig. 4, *E* and *F*). Our results suggest that the effect of deleting the ART motif is completely dependent on the single Phe116 residue, which is essential for β-arr2 recruitment to both class A and class B GPCRs. Notably, the association of β-arr2 with the V2R presents its strongest known interaction with GPCRs, as the V2R carboxyl tail is often appended to other GPCRs to stabilize β-arr binding. Hence, the loss of association of the F116A mutant with activated V2R further asserts the critical role of this residue in GPCR binding.

**Figure 5:**
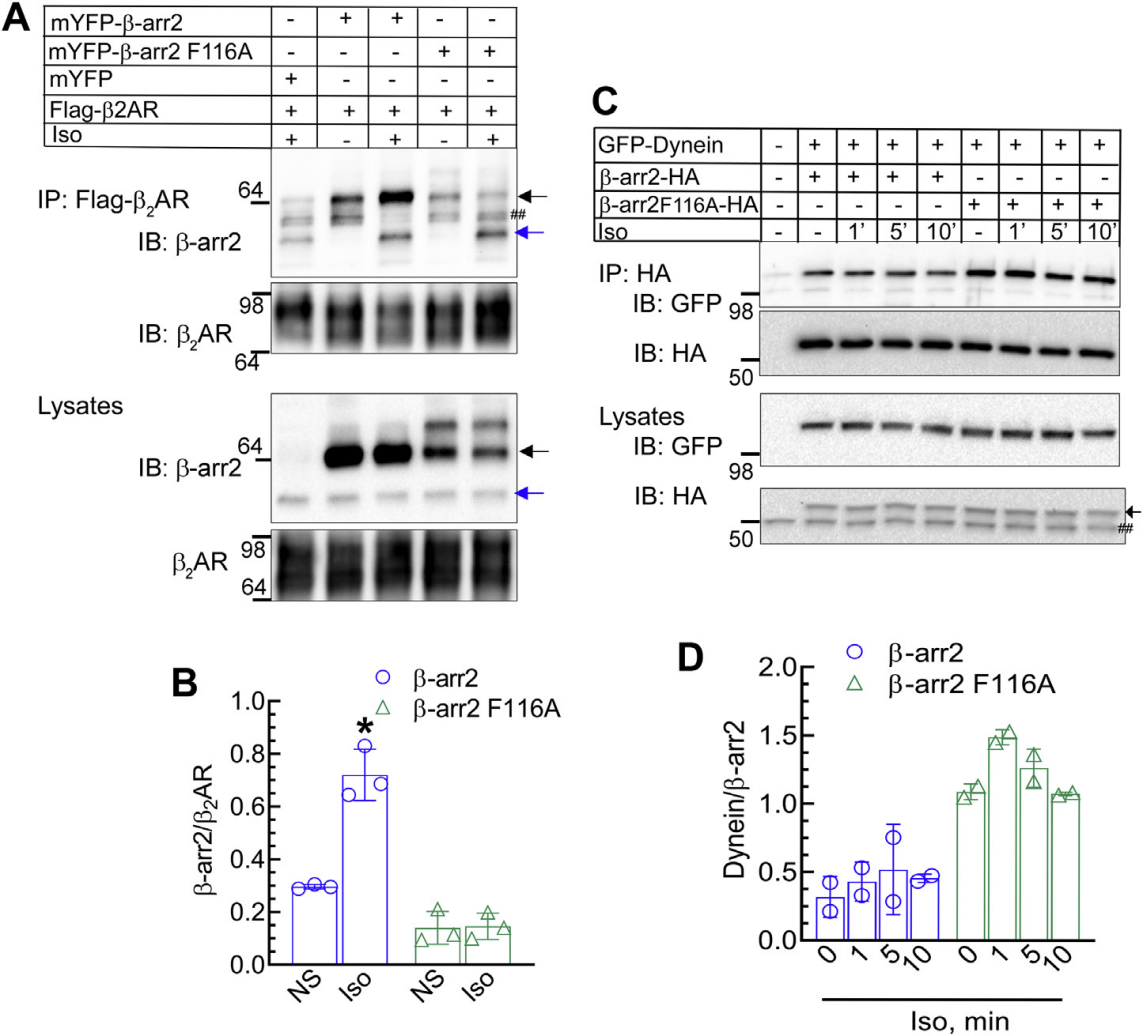
**GPCR and non-GPCR interactions of β-arr2F116A.***A*, HEK-293 cells stably expressing FLAG-β_2_AR were transfected with vector, YFP-β-arr2, or YFP-β-arr2F116A. After stimulation ± Iso for 10 min, FLAG receptors were immunoprecipitated after chemical crosslinking with DSP. The IPs and lysates were probed with a β-arr antibody (A1CT) and a FLAG M2 monoclonal antibody as indicated. ^##^Nonspecific bands. *B*, scatter plots with bars represents quantification of β-arr in the IP from three independent experiments and summarized as means ± SD. ∗*p* < 0.05 *versus* rest of the conditions, two-way ANOVA, and Holm–Sidak’s post test. *C*, HEK-293 cells stably transfected with β-arr2-HA or β-arr2 F116A-HA were transiently transfected with GFP-dynein, and β-arr2 immunoprecipitated by anti-HA affinity agarose beads were probed with a GFP antibody. Respective lysates were serially probed for GFP and HA tag in the lower two panels. ^##^Nonspecific band. *D*, dynein bands were normalized to cognate β-arr2 bands, and the ratio was plotted as means ± SD from two independent experiments. β_2_AR, β_2_-adrenergic receptor; β-arr2, β-arrestin2; DSP, dithiobis(succinimidyl propionate); GPCR, G protein–coupled receptor; HA, hemagglutinin; HEK-293, human embryonic kidney 293 cell line; IP, immunoprecipitation.

Our confocal imaging showed that the β-arr2F116A mutant failed to be recruited by the agonist-stimulated GPCRs. To confirm whether Phe116Ala mutation in β-arr2 completely ablated β-arr2's protein interaction with activated GPCRs, we performed coimmunoprecipitation (co-IP) assays. In our co-IP experiments, potential transient interaction of β-arr2 with the stimulated receptor was stabilized *via* chemical crosslinking using dithiobis(succinimidyl propionate) (DSP). As expected, after isoproterenol stimulation of FLAG-tagged β_2_AR, both endogenous and exogenous WT β-arr2 coimmunoprecipitated with the agonist-stimulated receptor (Fig. 5, *A* and *B*). On the other hand, chemical crosslinking failed to promote β-arr2F116A interaction with agonist-stimulated β_2_AR. In fact, agonist stimulation noticeably promoted co-IP of endogenous β-arr1 and β-arr2 (indicated with *blue arrow*, Fig. 5*A*) and not mutant β-arr2F116A (*black arrow*, Fig. 5*A*) with the activated receptor in the same immunocomplex pull down (Fig. 5*A*). This confirms that the Phe116Ala mutation impeded β-arr2 association with stimulated GPCRs. Since endogenous β-arrs could still bind to the β_2_AR in the same immunocomplex containing the β-arr2F116A mutant, it can be inferred that receptor activation and phosphorylation were still preserved and that the impaired function of the β-arr2F116A mutant was due to intrinsic disorder in the β-arr2F116A mutant protein and not from compromised receptor activation or phosphorylation.

Next, we determined whether mutation of Phe116 residue also affected β-arr2 interaction with its other binding partners. Co-IP experiments revealed that as with WT β-arr2, β-arr2F116A binding was preserved for the deubiquitinase USP20 and the E3 Ub ligase MDM2, which are known to promote the deubiquitination and ubiquitination of β-arr2, respectively (34, 39). Interestingly, β-arr interaction with its non-GPCR–binding partners was greater for the β-arr2F116A mutant compared with WT (Fig. S1, *A* and *B*). In addition, c-RAF, a component of β-arr–ERK signaling scaffold (44, 45), associated more robustly with β-arr2F116A than with WT (Fig. S1*C*). β-arr2 can bind several cytoskeletal proteins including cytoplasmic dynein (46), which is a microtubule-based motor that facilitates translocation and organization of cellular components. Both β-arr2 WT and β-arr2F116A associated with dynein intermediate chain IC2C (Fig. 5*C*). β-arr2 interaction with dynein subunit was not modulated by β_2_AR activation, and β-arr2F116A showed more robust association with dynein than WT β-arr2 (Fig. 5, *C* and *D*). Thus, while F116A mutation blocks the interaction of β-arr2 with GPCRs, the mutation perhaps locks β-arr2 in a conformation that is conducive for binding nonreceptor proteins.

**Figure 6:**
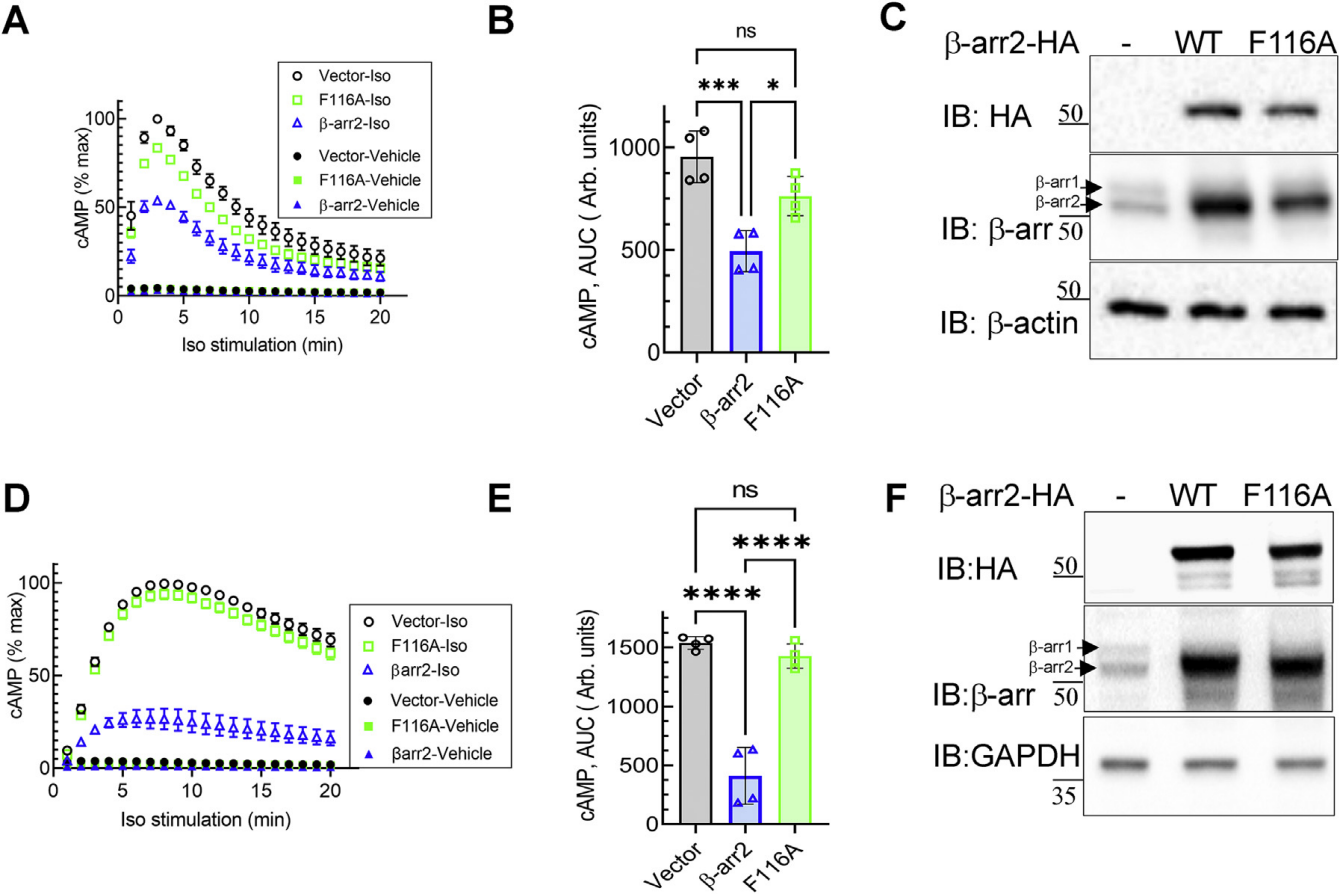
**Effects ofβ-arr2 and β-arr2F116A on β**_**2**_**AR-induced cAMP generation.** HEK-293 cells were transfected with vector, β-arr2-HA, or β-arr2F116A-HA along with pGloSensor (see the Experimental procedures section). The graph summarizes results from four independent experiments for cAMP detection in response to either vehicle or 100 nM isoproterenol for the indicated duration in the absence (*A*) or the presence (*D*) of 100 μM IBMX. *B* and *E*, cAMP calculated as area under the curve from the experiments shown in *A* and *D*, respectively, is presented as scatter plots with bars and ±SD. ∗*p*< 0.05, ∗∗∗*p* < 0.01, and ∗∗∗∗*p* < 0.0001 between indicated samples, one-way ANOVA, Tukey's post hoc comparison. *C* and *F*, whole cell extracts of aliquots of cells used in *A* and *D* were serially immunoblotted for HA, β-arr (A1CT), and β-actin or GAPDH. β_2_AR, β_2_-adrenergic receptor; β-arr2, β-arrestin2; HA, hemagglutinin; HEK-293, human embryonic kidney 293 cell line; IBMX, 3-isobutyl-1-methylxanthine.

Translocation of β-arr2 to the cell surface and interaction with the activated GPCR transmembrane core is essential for blocking G protein coupling and signal desensitization (17). The deficiency of recruitment observed for β-arr2F116A suggests an impairment in attenuating G protein activation as compared with WT β-arr2. To test this, we transfected HEK-293 cells with vector, β-arr2, or β-arr2F116A along with a cAMP biosensor (GloSensor; Promega) and monitored isoproterenol-induced cAMP generation by endogenous β_2_ARs. Although isoproterenol can also activate β_1_ARs, these HEK-293 cells express only β_2_AR endogenously and do not express any detectable β_1_AR (47). Isoproterenol stimulation of cells transfected with vector induced an increase in cAMP within 2 min, which declined to baseline within 20 min in the absence of inhibition of phosphodiesterases (PDEs), which rapidly degrade cAMP (Fig. 6*A*). Exogenous expression of β-arr2 blunted the maximal cAMP induced in cells transfected with control vector by ∼50 to 60% (Fig. 6, *A*–*C*), presenting desensitization. On the other hand, β-arr2F116A did not cause a significant decrease in cAMP as compared with vector conditions, but nonetheless caused an overall reduction of cAMP by ∼15% (Fig. 6, *A*–*C*). The difference between β-arr2 and β-arr2F116A in the blockade of cAMP generation became even more prominent when the assays were conducted in the presence of 3-isobutyl-1-methylxanthine (IBMX) that inhibits cellular PDEs. Upon inhibition of PDEs, overexpression of β-arr2F116A failed to decrease cAMP levels, which remained comparable to that of control vector conditions, in contrast to exogenous WT β-arr2, which significantly abated cAMP production (Fig. 6, *D*–*F*). These results confirm that β-arr2 interaction with activated β_2_AR is critical for desensitizing G protein signaling and that β-arr2F116A is defective in mediating this canonical function. Our data also suggest that despite its defect in associating with activated β_2_ARs, β-arr2F116A can yet slightly reduce cAMP levels, perhaps by its ability to scaffold PDE isoforms, or by mechanisms yet to be identified.

### Phe116 residue is essential for protein stability of β-arr2

**Figure 7:**
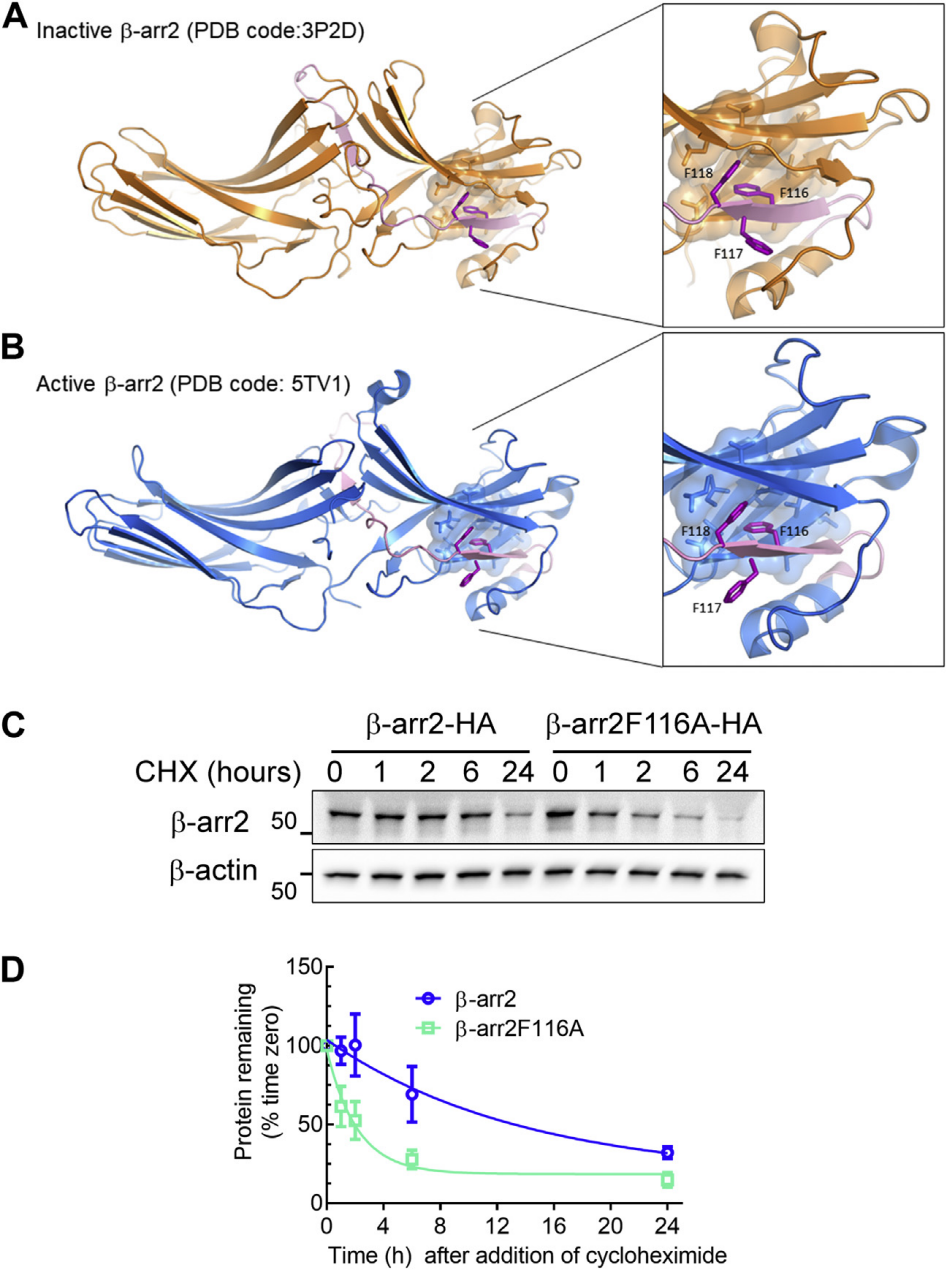
**Phe116 is located in a hydrophobic pocket of β-arrestin2 (β-arr2) and engenders protein stability of β-arr2.***A*, structural model of inactive β-arr2 (Protein Data Bank code: 3P2D) showing the position of the ART motif in *pink*. The enlarged section on the *right* shows the location of Phe116 in a hydrophobic pocket. *B*, structural model of active β-arr2 (Protein Data Bank code: 5TV1) showing the position of the ART motif in *pink*. The enlarged section on the *right* shows the location of Phe116, in the hydrophobic pocket shown in (*A*). In *A* and *B*, molecular figures were generated using PyMOL (DeLano, W. L. (2012) *The PyMOL Molecular Graphics System v. 1.3r1*, Schrödinger, LLC, New York, NY). *C*, HEK-293 cells stably transfected with β-arr2-HA or β-arr2 F116A-HA were treated with 50 μg/ml cycloheximide for indicated times, and whole cell extracts were immunoblotted serially for HA tag and β-actin. *D*, β-arr2 bands were normalized to cognate β-actin in each sample and summarized from five independent experiments as means ± SD. Protein half-lives determined by curve fitting (one-phase exponential decay) were 9.6 h (WT) and 1.5 h (F116A). ART, arrestin-related trafficking adaptor; HA, hemagglutinin; HEK-293, human embryonic kidney 293 cell line.

Next, we determined how the Phe116 residue of β-arr2 contributed to the structure and conformation of the protein, using available atomic structures of β-arr2. β-arrs are known to embrace distinct conformations, inactive and active, with the latter occurring upon the binding of β-arr to the phosphorylated tail of GPCRs (18, 48). Inactive β-arr2 structure (Protein Data Bank [PDB] code: 3P2D) revealed that the Phe116 residue was located with its side chain facing a hydrophobic patch consisting of Val21, Leu23, Val38, Val41, Val42, and Val56. Adjacent Phe117 residue is protruded away from the hydrophobic patch, whereas Phe118 is partially facing the hydrophobic patch (Fig. 7*A*). The inositol hexakisphosphate 6–activated β-arr2 (PDB code: 5TV1) showed no substantial rearrangement of Phe116, which still localized in the hydrophobic patch (Fig. 7*B*). Overall, the location of Phe116 proximal to a concentration of valine and leucine side chains suggests that Phe116 is ensconced in a hydrophobic pocket and that disruption of this structural arrangement affects conformational changes and/or protein stability of β-arr2.

We observed that the β-arr2F116A mutant presented reduced expression than WT with equivalent DNA transfections in cells, which suggested decreased protein stability resulting from the mutation. Thus, we investigated the turnover rate of the protein by performing cycloheximide (CHX)-chase assay. Exogenous β-arr2 WT had a significantly longer half-life compared with the β-arr2F116A mutant (9.5 h for WT β-arr2 *versus* 1.5 h for β-arr2F116A; Fig. 7, *C* and *D*). Since β-arr2F116A had a faster degradation rate compared with WT β-arr2, we investigated the mechanism involved by measuring the turnover rate of the β-arr2F116A mutant in the presence of the proteasome inhibitor MG132 or the lysosome inhibitor chloroquine. MG132 substantially rescued the degradation of β-arr2F116A in contrast to chloroquine, which failed to stabilize β-arr2F116A, indicating that the degradation of β-arr2F116A was mediated by the Ub proteasome system (Fig. S2). On the other hand, since addition of the lysosomal inhibitor chloroquine did not limit the degradation of β-arr2F116A, the turnover of β-arr2F116A was not mediated by the lysosomal pathway. Our results indicate that β-arr2F116A is less stable than WT β-arr2 and is targeted for degradation by the Ub–proteasomal system.

**Figure 8:**
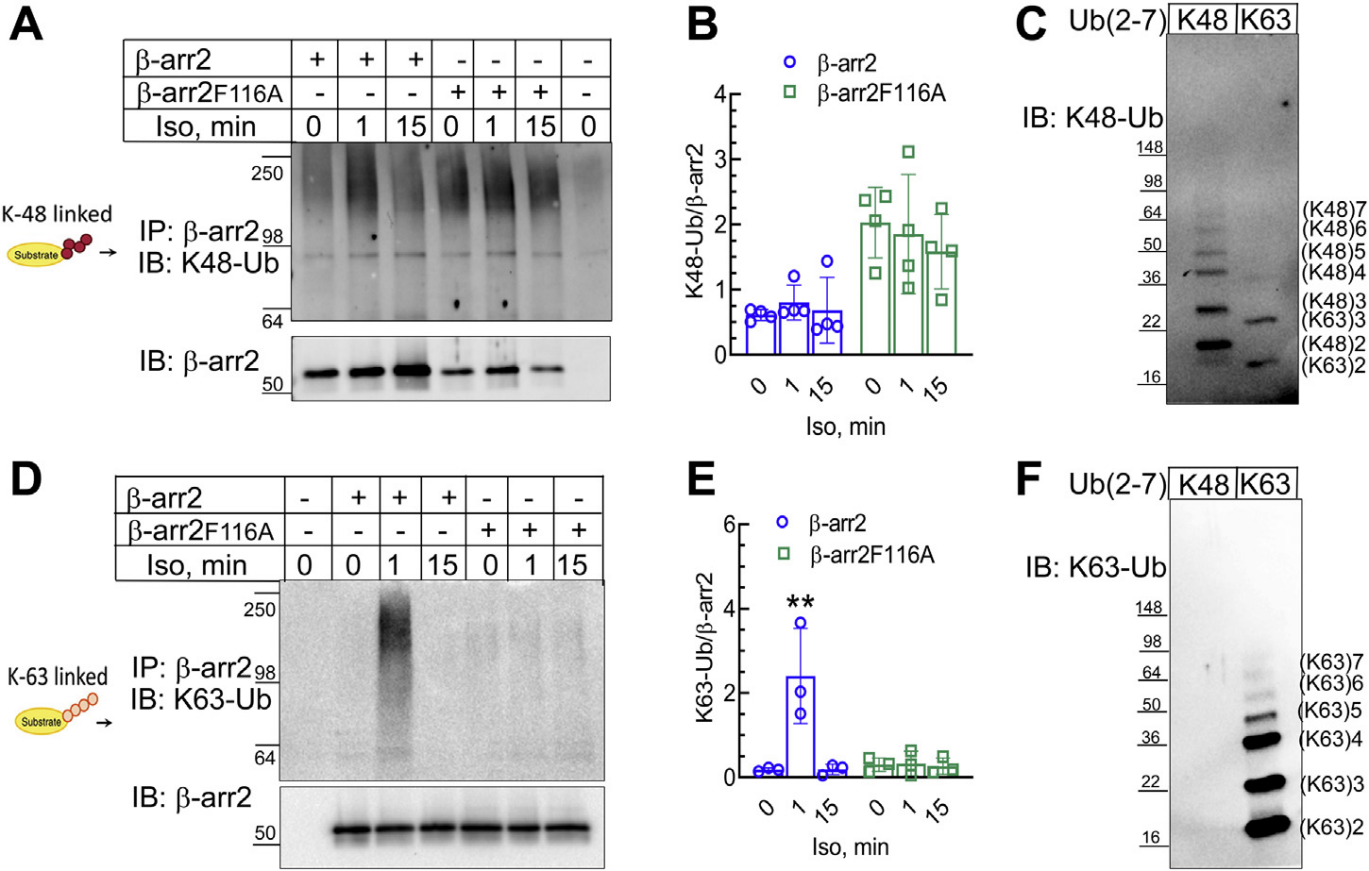
**Phe116 preserves β-arr2 conformation conducive for agonist-induced K63 polyubiquitination.***A*, HEK-293 cells were transfected with β-arr2-HA and β-arr2F116A-HA and stimulated ±1 μM Iso for the indicated times and β-arr2 was immunoprecipitated and probed with either IgG specific for K48-linked ubiquitin (Ub) or β-arr2. *B*, the Ub smears for each sample were normalized to cognate β-arr2 and plotted as bar graphs as means ± SD from four independent experiments. *C*, 1 μg of recombinant K48-linked His_6_-Ub chains (2, 3, 4, 5, 6, 7), and K63-linked untagged Ub chains (2, 3, 4, 5, 6, 7) were probed with the IgG specific for K48-linked Ub used in (*A*). *D*, experiments were conducted as in (*A*) except that an IgG specific for detecting K63-linked Ub was used. *E*, quantitation was conducted as in (*B*) and summarized from three independent experiments. ∗∗*p* < 0.01 *versus* all other conditions, two-way ANOVA and Holm–Sidak’s post test. *F*, experiment was carried out as in *C*, but the immunoblot was done with the IgG specific for detecting K63-linked Ub used in *D*. β-arr2, β-arrestin2; HA, hemagglutinin; HEK-293, human embryonic kidney 293 cell line; IgG, immunoglobulin G; K48, Lys-48; K63, Lys-63.

Proteins targeted for 26S proteasomal degradation present a specific Ub chain architecture encompassing attachment of additional Ub moieties to Lys 48 (K48) of Ub, resulting in distinctive polymeric Ub chain topology that is recognized by the proteasome (49). Since our CHX-chase experiments with MG132 revealed that β-arr2F116A turnover is mediated by the Ub–proteasome system, we investigated the ubiquitination architecture of WT β-arr2 and β-arr2F116A. Using an antibody that specifically recognizes K48 Ub chains (50), we investigated the ubiquitination profile of exogenous β-arr2 WT and β-arr2F116A mutant following β_2_AR agonist stimulation. Interestingly, β-arr2F116A presented substantial K48 polyubiquitination, which was approximately four times greater than WT β-arr2 ubiquitination (Fig. 8, *A* and *B*). Agonist stimulation did not engender a dramatic change over basal levels for K48 polyubiquitination of either WT β-arr2 or β-arr2F116A. However, substantial constitutive K48 polyubiquitination of β-arr2F116A suggests that the protein is steadily tagged for proteasomal degradation and explains the short half-life of the mutant protein. We also investigated the presence of Lys 63 (K63) polyubiquitin chains (50) in both β-arr2 WT and β-arr2F116A mutant induced by agonist stimulation of the β_2_AR. K63 polyubiquitin chains are mainly associated with protein scaffolding and signal transduction (49). Upon stimulation of the β_2_AR with isoproterenol, we observed a 10-fold increase of K63 polyubiquitination of exogenous β-arr2 WT at 1-min stimulation compared with basal levels (Fig. 8, *D* and *E*). On the other hand, isoproterenol stimulation failed to provoke K63 polyubiquitination of the β-arr2F116A mutant, for which ubiquitination remained at basal levels and was comparable to unstimulated β-arr2 WT. To determine the selectivity and specificity of the polyubiquitin chain immunoglobulins (IgGs), we tested their immunoreactivity toward 1 μg of recombinant Ub chains linked at K48 or K63 in Ub (Fig. 8, *C* and *F*). While the K63–Ub IgG had no detectable reactivity toward K48–Ub chains (Fig. 8*F*), we observed crossreactivity of K48–Ub IgG toward di-Ub and tri-Ub moieties but not with ≥4 Ub moieties with K63 linkage (Fig. 8*C*). Taken together, the absence of any signal for βarr2F116A with K63–Ub IgG confirms its defect in this type of modification, and the detection of WT β-arr2 by K48–Ub IgG likely presents both specific bands of K48 linkage and some cross-reactive bands that are K63 linked. Collectively, our data show that β_2_AR-agonist–triggered polyubiquitination of β-arr2 encompasses K63 polyubiquitin chains, which can facilitate scaffolding and signaling activities of β-arr2 when bound to the β_2_AR. The absence of K63 polyubiquitination in β-arr2F116A confirms that binding to the receptor is essential for this post-translational modification to occur and that the Phe116 residue dictates specific ubiquitination profiles of β-arr2, regulating the stability of β-arr2 and its interaction with activated GPCRs.

### Ub fusion of β-arr2 rescues the binding of β-arr2 to stimulated GPCRs

**Figure 9:**
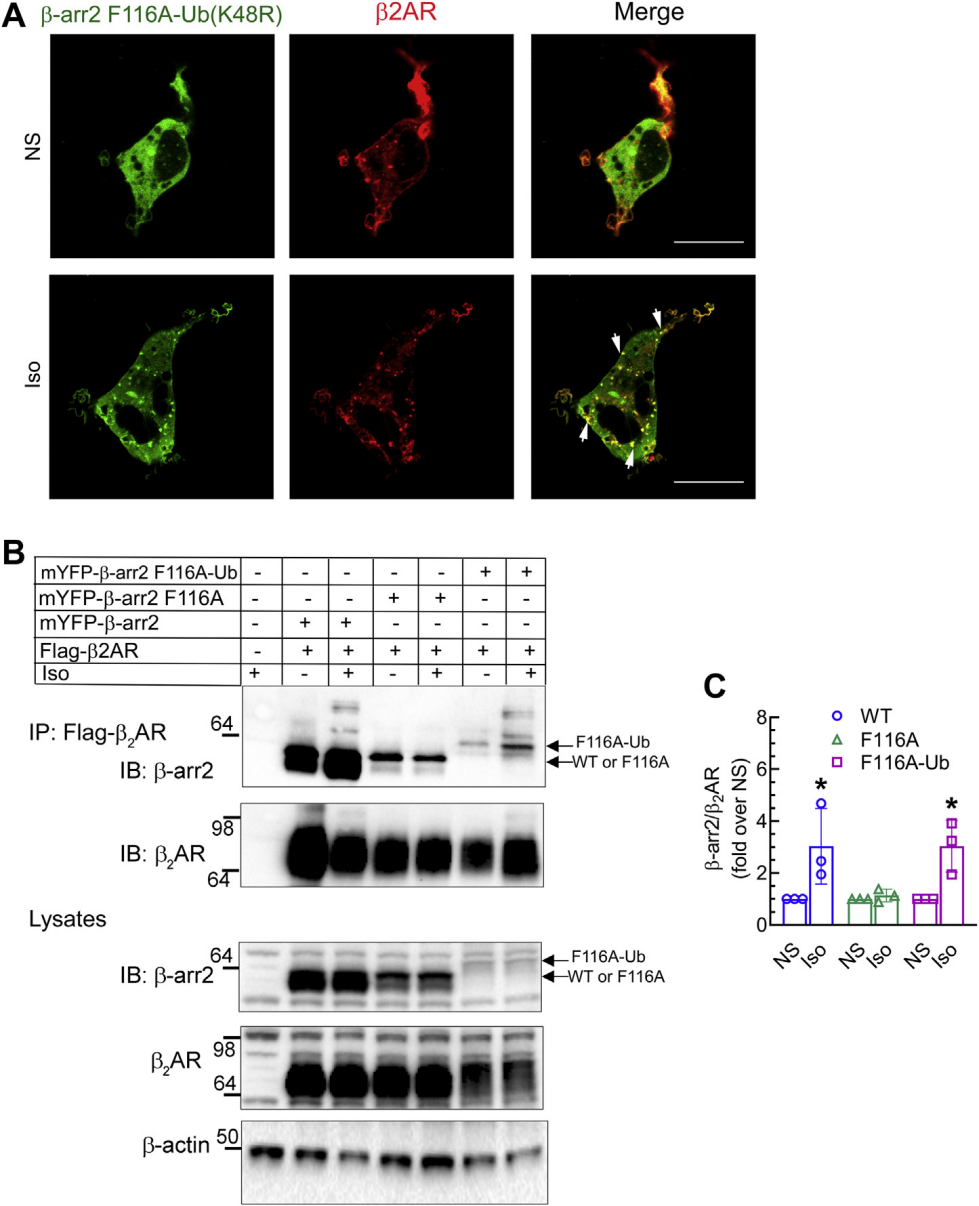
**Translational fusion of ubiquitin (Ub) rescues the defect in β-arr2F116A and facilitates strong binding with the β**_**2**_**AR.***A*, HEK-293 cells stably expressing β_2_AR, transfected with mYFP-β-arr2F116A-Ub (K48R) fusion protein were stimulated ±1 μM Iso for 15 min and subsequently processed for confocal imaging as described in the Experimental procedures section. Images from one of three separate experiments show distribution of β-arr2 F116A-Ub (K48R) in *green* and the β_2_AR in *red*. The scale bar represents 10 μm. *B*, HEK-293 cells stably expressing FLAG-β_2_AR were transfected with vector, YFP-βarr2, YFP-βarr2F116A, or YFP-βarr2F116A-Ub (K48R). After stimulation ± Iso for 15 min, and chemical crosslinking with DSP, FLAG receptors were immunoprecipitated. The IPs and lysates were serially probed with anti-GFP antibody that recognizes YFP (MBL International Corporation) to detect YFP-tagged β-arrs and a β_2_AR IgG (H-20; Santa Cruz Biotechnology) as indicated. Lysate blots were reprobed for β-actin. *C*, the bar graph represents quantification of β-arr in cognate β_2_AR IP with the values normalized to corresponding nonstimulated (NS) and plotted as means ± SD from three independent experiments. ∗*p* < 0.05 *versus* F116A stimulated samples and all NS samples, two-way ANOVA, and Holm–Sidak’s post test. β_2_AR, β_2_-adrenergic receptor; β-arr2, β-arrestin2; DSP, dithiobis(succinimidyl propionate); HEK-293, human embryonic kidney 293 cell line; IgG, immunoglobulin G; IP, immunoprecipitation.

Previous studies have shown that translational fusion of Ub to the C terminus of β-arr2 (β-arr2–Ub) enhances the binding of β-arr2 to β_2_AR, stabilizing receptor–β-arr2 complex formation, and conferring to that class A receptor, class B characteristics in regard to vesicular trafficking, and ERK signaling at endosomes (40, 42, 43, 51). Accordingly, we tested whether in-frame fusion of Ub to the β-arr2F116A mutant could rescue the recruitment of the mutant protein to the activated β_2_AR. To stabilize our chimeric β-arr2F116A-Ub protein and to limit its proteasomal degradation, we substituted K48 within the fused Ub to arginine. On the other hand, K63 chain formation within the fused Ub would be unaffected. Moreover, the Ub moiety in the chimeric mutant protein does not undergo deubiquitination by cellular deubiquitinases, permitting sustained β-arr2F116A ubiquitination. With confocal imaging, we assessed the recruitment of chimeric β-arr2F116A-Ub to the β_2_AR after agonist stimulation. Prior to agonist stimulation, β-arr2F116A-Ub mutant (*green*) was uniformly distributed in the cytoplasm (Fig. 9*A*). Agonist stimulation of β_2_AR with isoproterenol triggered the colocalization of β-arr2F116A-Ub (*green*) with the receptor (*red*) in vesicular compartments (Fig. 9*A*). Therefore, the Ub fusion stabilized the interaction of β-arr2F116A with β_2_AR, rendering to that type A GPCR, type B characteristics evidenced by the internalization, and vesicular trafficking of the receptor-β–arr2F116A–Ub complex. Concordantly, co-IP assays revealed that the association of β-arr2F116A-Ub with the β_2_AR was augmented by agonist stimulation, just as with WT β-arr2. On the other hand, β-arr2F116A showed basal association with the β_2_AR, which was unchanged with agonist stimulation (Fig. 9*B*). Taken together, our results show a cooperation between the ART motif and β-arr2 ubiquitination, with the former allowing receptor recognition by β-arr2 and the latter stabilizing the GPCR–β-arr2 complex, which altogether enable the signaling and trafficking activities of β-arrs.

## Discussion

In this study, we have defined the molecular role of the ART motif in β-arr2 with respect to GPCR association and have identified a residue, Phe116, in the ART motif that is important for the recruitment of β-arr2 to activated GPCRs. The binding of β-arr2 to the heptahelical transmembrane receptor is crucial for the receptor's subsequent signaling activities and β-arr-dependent signaling downstream of activated GPCRs. Targeting the ART motif, which enables receptor–β-arr interaction, may allow fine tuning the effects of β-arr on receptor desensitization or provide a handle for suppressing β-arr-mediated GPCR signaling.

The ART motif of β-arr is located in its N-terminal domain, which is a site of interaction of β-arr with GPCRs. Whether the ART motif directly associates with the GPCR domains is not evident from our studies here and would require further detailed structural studies. We employed available structures of inactive β-arr2 (23) and inositol hexakisphosphate 6–activated β-arr2 (52) to evaluate the structural features of ART motif in β-arr2. The inactive β-arr conformation is stabilized by a network of charged residues between the N and C domains in the fulcrum of the domain interface known as the polar core and by the three element interaction of β-strand I, α-helix I, and the carboxyl terminus of β-arr (24). The ART motif is not located in these subdomains of β-arr2, and our findings suggest that an additional region in β-arr contributes to its conformational architecture, protein stability, and GPCR association. Our study shows that the ART motif is not only essential for β-arr recruitment and binding to activated GPCRs but also important to enable agonist-mediated ubiquitination of β-arr2 and promote the stability of this multifunctional adaptor. Although we detected comparable protein expression and subcellular distribution for WT, ART motif–deleted and F116A β-arr2 constructs, whether the integrity of the arrestin fold of β-arr is preserved upon ablation of the ART motif or mutation of the Phe116 residue in β-arr2 requires a thorough assessment by structural characterization.

β-arr2 translocation to stimulated GPCRs is a necessary mechanism for the inhibition of G protein coupling and signal desensitization (17). While β-arr binding blocks β_2_AR-induced increase in cAMP, cellular levels of cAMP are also rapidly suppressed by PDEs (53, 54, 55). Although there was no detectable agonist-promoted binding of βarr2F116A with activated β_2_AR, we nonetheless observed a slight effect on cAMP reduction upon overexpression of this construct in HEK-293 cells. On the other hand, when we inhibited PDE activity, βarr2F116A overexpression did not lead to any detectable desensitization. Notably, βarr2 WT effectively blunted cAMP production in the presence and the absence of PDE inhibition. It is likely that the β-arr2F116A mutant, which displays robust binding with non-GPCR proteins, can promote cAMP degradation by scaffolding PDE (55). Elucidating how the ART motif regulates β-arr scaffolding of PDE isoforms and cAMP compartmentalization will provide further insights into the mechanisms that direct cyclic nucleotide activities and β-arr-mediated signal desensitization.

The recognition of phosphates on the activated GPCR by β-arr and binding to phosphorylated GPCRs are dependent on well-conserved lysine residues on the β-strand I of β-arr (43, 56). Interestingly, these lysine residues, which correspond to K11 and K12 in rat β-arr2, are also sites of agonist-triggered ubiquitination of β-arr, which stabilizes GPCR–β-arr complex (43). β_2_AR agonist–induced ubiquitination of β-arr2, which enhances the association of β-arr2 to the receptor, is contingent and not causative of the recruitment of β-arr2 to the activated receptor, because mutation of these lysine residues does not prevent β-arr2 binding to the receptor but weakens such interaction (43). Agonist-induced ubiquitination of β-arr2 was inhibited upon either ablation of the ART motif or mutation of the Phe116 residue of β-arr2 with alanine. Interestingly, our study showed that β-arr2 undergoes K63 polyubiquitination upon binding to the activated receptor. This specific Ub chain structure specializes in promoting protein–protein interaction and signal transduction as opposed to K48 polyubiquitination, which tags proteins for 26S proteasomal degradation. Our results suggest that the active conformation of β-arr, which is triggered upon binding to the phosphorylated tail of the receptor, involves K63 polyubiquitination. On the other hand, K48 polyubiquitination of the β-arr2F116A mutant occurs constitutively, targeting the protein for proteasomal degradation. In addition to inducing the degradation of the protein, it is likely that the architecture of K48 polyubiquitin chain interferes with the phosphate recognition of the lysine residues of β-arr2, impeding binding of β-arr2F116A mutant to the phosphorylated GPCRs. Assessing how specific Ub moieties delineate the function of β-arrs with regard to cell signaling may provide further insights into the signaling activities of these polyfunctional adaptors.

While the ART motif and Phe116 are critical for the association of β-arr2 and activated GPCRs, our pull-down assays revealed that the interaction of β-arr2 with several non-GPCR proteins remained unaffected or even augmented. The association of β-arr2 with the deubiquitinase USP20 and the E3 ligases MDM2 and TRAF6 were preserved despite deletion of the ART motif and/or the Phe116Ala mutation. Indeed, some of these interactions of β-arr2 have been shown to be enhanced by GPCR agonists. On the other hand, the same protein interactions of β-arr2 may or may not require GPCR activation depending on the cellular pathway involved. For example, while MDM2 ubiquitinates β-arrs in an agonist-dependent manner to stabilize GPCR–β-arr complex and promote β-arr scaffolding of components of the endocytic machineries and MAPK cascade (14), the partnership between β-arr and MDM2 is not limited to GPCRs. Indeed, β-arr2, which contains a nuclear export motif in its C terminus, interacts with MDM2 independently of GPCRs and mediates MDM2's nuclear export and relocation in the cytoplasm (57). β-arr2 also associates with MDM2 to facilitate the ubiquitination of various cytoplasmic proteins such as GPCR kinase 2 and the androgen receptor, which are subsequently targeted for proteasomal degradation (16, 58, 59, 60). Whether the ART motif in β-arr2 is involved in facilitating ubiquitination of other MDM2 substrates remains to be defined.

Besides MDM2, β-arr2 serves as an adaptor for a variety of Ub ligases, such as TRAF6, members of the HECT domain family NEDD4, SMAD ubiquitination regulatory factor-2, carboxyl terminus of Hsc70-interacting protein, atrophin-1 interacting protein 4/ITCH, NEDD4L, components of the Cullin-RING–Ub–ligase complex KBTBD8 and Kelch-like 12, as well as Parkin, an E3 ligase of the RING-in-between RING family (16, 61, 62, 63). It is unknown whether deletion of the ART motif transforms β-arr2 into a substrate for the aforementioned ubiquitinating enzymes or whether a conformational change in the β-arr2 mutant protein leads to recognition and ubiquitination of β-arr2 by novel Ub ligases to promote its proteasomal degradation.

The sequence alignment of β-arr2 and yeast Art1 revealed that many residues are conserved in the ART motif. In yeast Art1, Gly193 (corresponding to β-arr2 Gly110) was shown to be critical for Art1 function in the endocytic downregulation of plasma membrane proteins (28). However, Phe116 and not Gly110 in β-arr2 is a critical residue in the ART motif to enable β-arr recruitment to stimulated GPCRs. Phenylalanine, which is a nonpolar aromatic amino acid, is known to promote interaction with nonpolar residues and stabilize interaction of proteins with the plasma membrane (64, 65). Phe116 is also conserved in β-arr1; the role of the ART motif in β-arr1 although expected to function as in β-arr2, is undefined and requires further studies. The other conserved residues in the ART motif while not contributing to receptor–β-arr2 interaction may facilitate additional functions of β-arr2 as the multifunctional adaptor interacts with a variety of receptor families, as well as several membrane and cytoplasmic proteins to regulate various cellular activities (2).

Mutation of the Phe116 residue in β-arr2 to alanine augmented the binding of β-arr2 to cytoplasmic dynein, which is one of the motor proteins identified in interactome studies (46). Moreover, the interaction of β-arr2 with c-RAF was promoted upon mutation of Phe116 in the ART motif with alanine. c-RAF, which usually prefers GPCR-bound β-arr, is an essential component of the c-Raf1–MEK1–ERK1/2 cascade, enabling β-arr-dependent GPCR-induced MAPK activation. Nevertheless, while ablation of the ART motif may disallow GPCR-triggered/β-arr2-mediated ERK signaling by preventing GPCR–β-arr complex formation, the overall scaffolding properties of β-arr2 are retained in the β-arr2ΔART and β-arr2F116A mutants. β-arr2 is known to promote specific MAPK pathways such as the apoptosis signal–regulating kinase 1–/mitogen-activated kinase kinase 4–/c-Jun N-terminal kinase 3 (ASK1-MKK4-JNK3) cascade, independent of GPCR binding (66, 67); therefore, even though β-arr2F116A mutant is impaired in GPCR interaction, it may still allow ERK or JNK3 activation in suitable cellular conditions (66). Furthermore, because β-arr2F116A does not effectively block G protein signaling, MAPK signaling that is mediated by G protein activity may be potentiated in cells overexpressing this mutant (68).

We showed that chimeric β-arr2 fusion with Ub covalently attached to its carboxyl terminus rescued the recruitment of β-arr2F116A to activated β_2_AR. β-arr2 ubiquitination, which occurs after agonist stimulation of either type A or type B GPCRs, as well as for tyrosine kinase receptors like the insulin-like growth factor 1 receptor, and the pattern recognition receptor Toll-like receptor 4, enable various functions of β-arr such as receptor vesicular trafficking, protein scaffolding, and signal transduction (12). While ubiquitination of β-arr2 is provoked or reversed by E3 Ub ligases and deubiquitinases, respectively, it is a dynamic process that dictates the kinetics of GPCR–β-arr interaction. In contrast, ubiquitination in the chimeric β-arr2–Ub fusion protein is more stable as the chimera does not undergo deubiquitination by cellular deubiquitinases (12, 42). The rescue of β-arr2F116A recruitment and stabilization of receptor–β-arr2F116A interaction by the Ub fusion highlight the role of Ub in strengthening GPCR–β-arr interaction and in promoting vesicular trafficking of the receptor. In summary, our study provides novel insights into mechanisms that regulate the functions of β-arr in GPCR signaling, revealing a cooperative role between the ART motif and β-arr ubiquitination in promoting β-arr translocation to the cell surface and stabilizing GPCR–β-arr complex formation. Our findings also provide additional tools and approaches to regulate the kinetics of GPCR–β-arr2 interaction, which may lead to further breakthroughs in GPCR pharmacology.

## Experimental procedures

### Cell lines

HEK-293 cells and African green monkey kidney fibroblast–like cell line COS-7 were purchased from the American Type Culture Collection. HEK-293 cells were grown in minimal essential medium, whereas the COS-7 cells were maintained in Dulbecco's modified Eagle's medium. Growth media were supplemented with 10% fetal bovine serum, 1% penicillin–streptomycin, and kept at 37 °C in a humidified incubator containing 5% CO_2_. Plasmid transfections in these cells were performed with Lipofectamine 2000 (Thermo Fisher Scientific) following manufacturer’s protocol. Selection of stable HEK-293 cell lines expressing hemagglutinin (HA)-tagged WT β-arr2, β-arr2ΔART, or β-arr2F116A was achieved by supplementing growth medium with 1 mg/ml of G418, which was later lowered to 400 μg/ml for cell maintenance, as described previously (34). HEK-293 cells with stable expression of AT_1a_R or V2R were generated by using zeocin selection (200 μg/ml), and selected stable cells were maintained at 75 μg/ml of zeocin. Stable cell lines with dual expression of exogenous FLAG-β_2_AR and mYFP-tagged β-arr2, β-arr2ΔART, β-arr2F116A, or β-arr2F116A-Ub were generated by sequential clone selection with hygromycin b gold (InvivoGen; 150 μg/ml), followed by G418 (1 mg/ml). The cells were later maintained in growth medium containing 25 μg/ml of hygromycin b gold and 400 μg/ml of G418.

### Reagents

(-)-Isoproterenol (catalog no.: I2760), AngII (catalog no.: 4474-91-3), [Arg8]-Vasopressin acetate salt, V9879, anti-HA affinity gel (catalog no.: E6779), M2 anti-FLAG affinity-agarose beads (catalog no.: A2220), MG132 (catalog no.: C2211), *N*-ethylmaleimide (catalog no.: E1271), 3-isobutyl-1-methylxanthine IBMX (catalog no.: I5879), and Triton X-100 (catalog no.: T-9284) were purchased from Sigma–Aldrich. Chloroquine (catalog no.: 193919) was obtained from MP Biomedicals, CHX (catalog no.: 66-81-9) from Calbiochem, and DSP (catalog no.: PG82081) from Pierce. Recombinant human poly-Ub WT chains (2, 3, 4, 5, 6, 7) (K63) (catalog no.: UC330) and recombinant human His6 poly-Ub WT chains (2, 3, 4, 5, 6, 7) (K48) (catalog no.: UCH230) were purchased from Boston Biochem. The molecular weights for K63-chain components are 17 kDa (Ub2), 26 kDa (Ub3), 34 kDa (Ub4), 43 kDa (Ub5), 52 kDa (Ub6), and 60 kDa (Ub7), whereas the molecular weights of K48-chain components are 19 kDa (Ub2), 29 kDa (Ub3), 38 kDa (Ub4), 48 kDa (Ub5), 58 kDa (Ub6), and 67 kDa (Ub7).

### Antibodies

The antibodies used for our studies were the following: anti-β-actin (catalog no.: A5441) and anti-FLAG M2 (catalog no.: F3165) from Sigma–Aldrich; anti-β_2_AR H-20 (catalog no.: sc-569), anti-HA (catalog no.: sc-805), anti-c-RAF (catalog no.: sc-133), and anti-TRAF6 (catalog no.: sc-7221) from Santa Cruz Biotechnology; anti-HA 12CA5 (catalog no.: 11666606001) from Roche diagnostics; anti-Ub FK2-horseradish peroxidase (HRP) (catalog no.: BML-PW0150) from Enzo Life Sciences; anti-K63-linked polyubiquitin (clone APU3; catalog no.: 05-1308), from Millipore; anti-K48-linked polyubiquitin (catalog no.: 12805) and anti-GAPDH-HRP (catalog no.: 3683) from Cell Signaling Technology; anti-USP20 (catalog no.: A301-189A) from Bethyl Laboratories, Inc; anti-MDM2 (catalog no.: ab87134) from Abcam, Inc; and anti-GFP/GFP variants (catalog no.: MBL-598) from MBL International Corporation. Secondary antibodies conjugated to HRP were obtained from GE Healthcare or Rockland Immunochemicals, whereas secondary antibodies conjugated to Alexa fluorophores were purchased from Invitrogen. Anti-β-arr antibodies A1CT and A2CT were kindly provided by Dr Robert J. Lefkowitz (Duke University, Durham, NC).

### Plasmids

A plasmid encoding dynein intermediate-chain IC2C was purchased from Addgene (catalog no.: 51409) (69). Plasmids encoding rat β-arr2, Mdm2, USP20, and c-Raf-1 were described in earlier studies (14, 40, 43). Deletion of the ART motif was accomplished by using Q5 Site-directed Mutagenesis Protocol (NEB), whereas the mutations of individual amino acid residues in the ART motif to alanine were achieved using the QuikChange site-directed mutagenesis kit (Stratagene) following the manufacturers’ protocol and using WT β-arr2 plasmid as a template. YFP-tagged β-arr2 was modified by C-terminal in-frame fusion with Ub K48R as described previously (43). All plasmid constructs were confirmed by DNA sequencing.

### CHX-chase experiments

Stable cell lines expressing either exogenous HA-tagged β-arr2 or β-arr2F116A were treated with 50 μg/ml CHX to inhibit protein synthesis and harvested at the indicated time points to determine β-arr levels and degradation rate. Investigation of the degradation pathways taken by the β-arr2F116A mutant was done by adding to the corresponding cells either the 26S proteasomal inhibitor MG132 (10 μM) or the lysosomal inhibitor chloroquine (25 μM) 30 min before CHX addition. Cells were harvested at the indicated time point in 2× SDS sample buffer and processed for immunoblotting as described previously (34).

### Chemical crosslinking

Analysis of β_2_AR–β-arr2 interactions was performed by utilizing in-cell chemical crosslinking with the membrane-permeable lysine-reactive crosslinker DSP as described previously (70). HEK-293 cells with dual stable expression of exogenous FLAG-β_2_AR and YFP-tagged β-arr2 WT or Phe116Ala mutant were plated on poly-d-lysine–coated dishes of 100 mm and maintained at 37 °C until they reached >80% confluence. The cells were then treated with agonist or vehicle at 37 °C for the indicated time, and cell stimulation was terminated by incubating the cells with the cross-linker solution for 20 min at room temperature in a rotating shaker. Then, the chemical cross-linking reaction was quenched by adding Tris–HCl, pH 7.5 (final concentration of 25 mM), followed by three washes with PBS containing 10 mM 4-(2-hydroxyethyl)-1-piperazineethanesulfonic acid (HEPES) (pH 7.5) for removal of unreacted/excess DSP. Cell lysates were prepared using radioimmunoprecipitation assay buffer (150 mM NaCl, 50 mM Tris, pH 8.0, 5 mM EDTA, 1% Nonidet P-40 [NP-40], and 0.5% deoxycholate) supplemented with protease inhibitors and receptors immunoprecipitated as described later.

### IP and immunoblotting

IP and immunoblotting were performed as described previously (34). Briefly, cells were starved for 1 h in serum-free medium and stimulated with agonist or vehicle at the indicated time. Receptor stimulation was terminated by washing the cells with ice-cold PBS (pH 7.4) followed by lysis in ice-cold lysis buffer containing 50 mM HEPES, pH 7.5, 2 mM EDTA, 250 mM NaCl, 10% (v/v) glycerol, 0.5% NP-40 and supplemented with phosphatase and protease inhibitors (1 mM sodium orthovanadate, 10 mM sodium fluoride, 100 μM phenylmethylsulfonyl fluoride, leupeptin [5 μg/ml], aprotinin [5 μg/ml], pepstatin A [1 μg/ml], and benzaminidine [1 mM]). Cell lysates were then centrifuged at 15,000*g* for 30 min at 4 °C, and the supernatant was collected. After assessment of protein concentrations *via* Bradford protein assay, equivalent proteins were used for IP and/or immunoblotting. IP was done with either anti-FLAG M2 resin or anti-HA agarose beads that were added to the cell extracts, which were then incubated at 4 °C overnight with end-over-end rotation. After overnight incubation, immunocomplexes were washed with radioimmunoprecipitation assay or NP-40 lysis buffer, and bound proteins were eluted in 1× SDS-PAGE sample buffer. Protein samples (immunocomplexes and corresponding whole cell extracts) were then resolved by SDS-PAGE using either 4 to 20% gradient gels or 10% gels (Invitrogen). Resolved proteins were then transferred onto a nitrocellulose membrane for immunoblotting. Membranes were blocked in 25 mM Tris, pH 8.0, 150 mM NaCl, and 0.1% Tween-20 buffer supplemented with 5% (w/v) dried skim milk powder. Antibody incubations were performed in blocking solution, with in-between washes in 25 mM Tris, pH 8.0, 150 mM NaCl, 0.1% Tween-20 buffer. Immunoreactive bands were visualized using enhanced chemiluminescence (SuperSignal West Pico Reagent; Pierce) for light emission, a charged-coupled device camera system (Bio-Rad; Chemidoc-XRS) for signal detection and image acquisition and Image-Lab software (Bio-Rad) for data analysis. After immunoblotting, the blots were stripped with a Western blot stripping solution and reprobed with specified loading control antibodies to confirm equal loading.

### Measurements of cAMP production

The level of cAMP was determined using Glosensor, a chemiluminescence-based cAMP biosensor (Promega (71)). HEK-293 cells seeded on 6-well dishes at 80% confluence were transfected with Lipofectamine 2000 with either vector, β-arr2-HA, or β-arr2F116A-HA along with p-GloSensor-22F plasmid. Parallel transfections were set up in 6-well dishes, and cells were used at the experiment end point for preparing extracts that were subjected to SDS-PAGE and Western blot analysis to detect β-arr2 expression. For the cAMP assay, cells were detached 4 h post-transfection resuspended in clear minimal essential medium containing 2% fetal bovine serum + 1% penicillin–streptomycin + 10 mM HEPES and reseeded in 96-well white clear bottomed plates that were previously coated with poly-d-lysine. About 18 to 20 h later, cells were washed with Hanks' balanced salt solution (HBSS), GloSensor reagent diluted in HBSS was added, and incubation was continued for 1 h at 26 °C. Subsequently, GloSensor reagent was replaced with 90 μl of HBSS supplemented with 10 mM HEPES, pH 7.5, and plates were subjected to a baseline preread for luminescence on a Synergy Neo2 plate reader driven by Gen5 Software (BioTek Instruments). Next 10 μl of vehicle or agonist isoproterenol at desired concentration was added to respective wells, and the plates were immediately read for luminescence at 26 °C. For cAMP PDE inhibition, cells were incubated with 100 μM IBMX 15 min before agonist addition.

### Immunofluorescence staining and confocal imaging

Immunofluorescence staining and confocal imaging were performed as described previously (51, 72, 73). HEK-293 cells stably expressing exogenous FLAG-β_2_AR or HA-AT_1a_R, or HA-V2R and mYFP-tagged β-arr2, β-arr2ΔART, β-arr2F116A, or β-arr2F116A-Ub were plated on poly-d-lysine–coated 35-mm glass bottom plates (MatTek Corp). About 24 h later, the cells were serum starved for 1 h and stimulated with corresponding GPCR agonists for the indicated time. After stimulation, cells were fixed by incubating them for 15 min in 5% formaldehyde diluted in calcium/magnesium-containing Dulbecco's PBS (DPBS). Cells were then permeabilized by incubating them for 20 min at room temperature in 0.1% of Triton X-100 diluted in DPBS containing 2% bovine serum albumin. After two washes with DPBS, the cells were incubated with appropriate antibodies diluted in DPBS containing 2% bovine serum albumin, with overnight incubation at 4 °C for primary antibody, followed by four washes in DPBS, and 1 h incubation at room temperature for corresponding secondary antibody. After three washes with DPBS, cells were visualized with LSM-510 META confocal microscope with filter settings for respective fluorophores: excitation/emission (488 nm/515–540 nm, Alexa 488; 568 nm/585–615 nm, Alexa 594). Multicolor images were acquired by the LSM operating software (ZEISS ZEN imaging software) in the sequential acquisition mode to prevent crossexcitation. All assays were repeated, and observations were confirmed in at least three independent experiments.

### Statistical analysis

Data from at least three independent experiments were averaged and represented as means ± SD. Statistical analyses were performed with ANOVA followed by post hoc correction for multiple comparisons using statistical analysis software, GraphPad Prism 9 (GraphPad Software, Inc). Significance was defined as *p* < 0.05.

## Data availability

All data, associated methods, and sources of materials are available in the main text or in the supporting information.

## Supporting information

This article contains supporting information.

## Conflict of interest

The authors declare that they have no conflicts of interest with the contents of this article.
