# Supplementary material for: A single phenylalanine residue in β-arrestin2 critically regulates its binding to G protein–coupled receptors
Source: J Biol Chem. 2022 Mar 17;298(5):101837. doi: 10.1016/j.jbc.2022.101837 (PMC9052155; doi:10.1016/j.jbc.2022.101837)
Supplement: Supplemental Figures S1 and S2 [file mmc1.pdf]

A single phenylalanine residue in β-arrestin2 critically regulates its binding to G protein-coupled receptors

Pierre-Yves Jean-Charles[1,#], Vishwaesh Rajiv[1], Subhodeep Sarker[1,§], Sangoh Han[1], Yushi Bai[1], Ali Masoudi[1], and Sudha K. Shenoy[1,2,3]

SUPPORTING INFORMATION

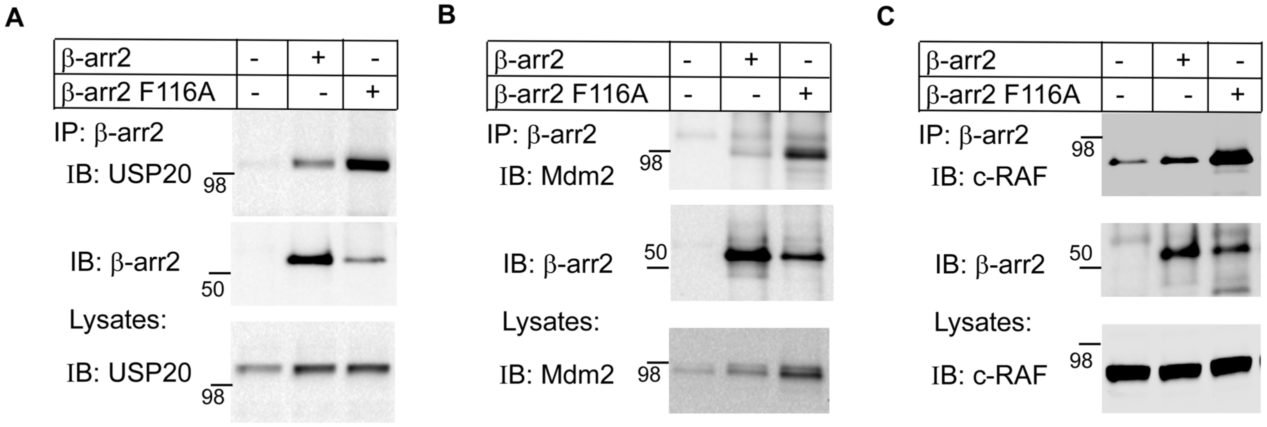

Figure S1. Protein-protein interactions of β-arrestin2 F116A mutant. COS-7 cells were transfected with vector, β-arr2-Flag, or β-arr2F116A-Flag along with plasmids encoding USP20 (A), Mdm2 (B) or c-RAF (C). Flag immunoprecipitates were serially probed for USP20 (A), Mdm2 (B) or c-RAF (C) followed by A2CT (to detect β-arr2). Expression of USP20, Mdm2 and c-RAF in lysates are displayed in the lowest panels. The blots shown are representative of one from three independent experiments.

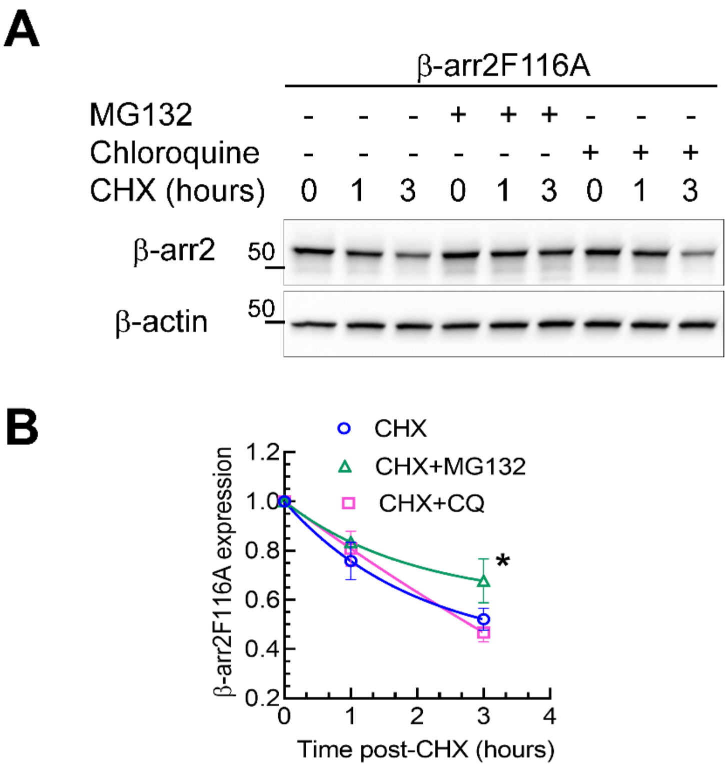

Figure S2. β-arrestin2 F116A undergoes rapid degradation via the 26S proteasomal machinery.

A) HEK-293 cells over-expressing HA-tagged β-arr2F116A were treated with CHX alone or along with MG132 or chloroquine for 0, 1, and 3 hours. Whole cell lysates were immunoblotted with anti-HA IgG (Cell Signaling) and β-actin (Sigma) sequentially.

B) Line graphs representing relative levels of β-arr2F116A following the treatments described in (A). β-arr2F116A was normalized to β-actin and represented as means ± SD. Degradation rate of β-arr2F116A after the addition of CHX was significantly reduced in the presence of MG132 and not affected by chloroquine. N = 3, * $p < 0.05$, versus all other samples at 3 hours, Two-way ANOVA, Holm-Sidak's posttest.
